# Optogenetic Mimicry of the Transient Activation of Dopamine Neurons by Natural Reward Is Sufficient for Operant Reinforcement

**DOI:** 10.1371/journal.pone.0033612

**Published:** 2012-04-10

**Authors:** Kyung Man Kim, Michael V. Baratta, Aimei Yang, Doheon Lee, Edward S. Boyden, Christopher D. Fiorillo

**Affiliations:** 1 Department of Bio and Brain Engineering, Korea Advanced Institute of Science and Technology, Daejeon, South Korea; 2 Media Lab, McGovern Institute, and Biological Engineering, Massachusetts Institute of Technology, Cambridge, Massachusetts, United States of America; University of Chicago, United States of America

## Abstract

Activation of dopamine receptors in forebrain regions, for minutes or longer, is known to be sufficient for positive reinforcement of stimuli and actions. However, the firing rate of dopamine neurons is increased for only about 200 milliseconds following natural reward events that are better than expected, a response which has been described as a “reward prediction error” (RPE). Although RPE drives reinforcement learning (RL) in computational models, it has not been possible to directly test whether the transient dopamine signal actually drives RL. Here we have performed optical stimulation of genetically targeted ventral tegmental area (VTA) dopamine neurons expressing Channelrhodopsin-2 (ChR2) in mice. We mimicked the transient activation of dopamine neurons that occurs in response to natural reward by applying a light pulse of 200 ms in VTA. When a single light pulse followed each self-initiated nose poke, it was sufficient in itself to cause operant reinforcement. Furthermore, when optical stimulation was delivered in separate sessions according to a predetermined pattern, it increased locomotion and contralateral rotations, behaviors that are known to result from activation of dopamine neurons. All three of the optically induced operant and locomotor behaviors were tightly correlated with the number of VTA dopamine neurons that expressed ChR2, providing additional evidence that the behavioral responses were caused by activation of dopamine neurons. These results provide strong evidence that the transient activation of dopamine neurons provides a functional reward signal that drives learning, in support of RL theories of dopamine function.

## Introduction

There is extensive evidence, particularly from pharmacological studies, that dopamine can cause positive reinforcement of stimuli and actions [1], [2]. Furthermore, electrophysiological recordings in behaving animals demonstrate that the firing rate of midbrain dopamine neurons of VTA and substantia nigra (SN) is elevated by natural reward events for about 200 ms. A large body of evidence indicates that this transient response is aptly described as a “reward prediction error” (RPE), since firing rate increases in response to events that are better than predicted, decreases in response to events that are worse than predicted, and undergoes little change in response to events that meet expectations [3]–[10].

RPE drives reinforcement in computational models of RL [11], [12], and thus it has been proposed that dopamine could serve an analogous reinforcing function in the brain [13], [14]. The RL theory of dopamine function has been highly influential, and if correct, it would unify a large body of research. However, compelling evidence in favor of the theory has been lacking, and the theory has been criticized on a variety of grounds.

First, although the transient response of dopamine neurons has the appropriate properties to drive RL from a computational perspective, there is little direct evidence that it actually does. Virtually all the direct evidence that dopamine causes reinforcement has come from manipulations that modulate dopamine for periods of at least minutes or longer [1], [2], [15], [16]. By contrast, natural reward events increase the firing rate of dopamine neurons for only about 0.2 s, causing a transient increase in dopamine concentration lasting 1–2 s [17]. Second, some have proposed that the transient dopamine signal does not actually represent reward, and thus may not be appropriate for a positive reinforcement signal [18], [19], but see [20]. This viewpoint has been based in part on the argument that the increased firing rate of dopamine neurons occurs too soon after onset of a stimulus (∼100–200 ms) for an accurate assessment of whether or not it is rewarding [19]. In addition, there is evidence that under some conditions dopamine neurons can be activated by physically salient events that may not have reward value [18], [21], [22]. Finally, reinforcement is a process of associative learning, and it has been argued that dopamine does not directly cause associative learning [23], [24]. Consistent with this argument, it has been demonstrated that dopamine is not necessary for all reinforcement learning [24]–[26].

It has not previously been possible to directly test for a causal role of the transient dopamine signal in reinforcement. Pharmacological and other manipulations act on a much slower timescale, whereas electrical stimulation is not able to selectively activate dopamine neurons. However, it should now be possible to test the RL theory of dopamine function in a direct and definitive manner by using optogenetic techniques, which allow temporal control of neurons with a precision in the range of milliseconds, together with the ability to target genetically defined neuronal populations [27]. Optogenetics has previously been used to selectively target dopamine neurons in mice [16], [28]–[32].

Tsai and colleagues first demonstrated the feasibility of optogenetic stimulation of dopamine neurons in mice [16]. They showed that brief optical stimulation of the VTA could elevate the firing rate of dopamine neurons both in vivo and in vitro (as measured by electrophysiology). Furthermore, a 0.5 s train of optical stimuli evoked an increase in dopamine concentration in the nucleus accumbens (as measured by cyclic voltametry) that lasted for several seconds [16], similar in duration to the transient rise in dopamine that has been observed previously in response to natural rewards [17].

It is critical to note that the study of Tsai and colleagues [16] did not directly test the role of the transient dopamine signal in reinforcement. What they showed was that repeated optical activation of dopamine neurons over a period of 30 minutes was sufficient to induce a conditioned place preference, a form of Pavlovian conditioning. Thus the “conditioned stimulus” (provided by the environment) was 30 minutes in duration, and the “unconditioned stimulus” (provided by the dopamine) extended over the same 30 minute period (although it was fluctuating “phasically” throughout that time). Their results confirmed the conclusions of previous studies showing that drugs that activate dopamine receptors, over minutes or longer, are sufficient for positive reinforcement (e.g. [1], [2], [15]). However, the RL theory of dopamine function proposes a much more temporally precise and computationally powerful role for dopamine neurons. Specifically, the RL theory proposes that a stimulus or action occurring at one moment will be reinforced if a single, transient activation of dopamine neurons occurs just a fraction of a second later (with weaker reinforcement in the case of longer delays). The simple goal of the present study is to test the hypothesis that the transient activation of dopamine neurons is sufficient in itself to drive operant reinforcement.

## Results

**Figure 1:**
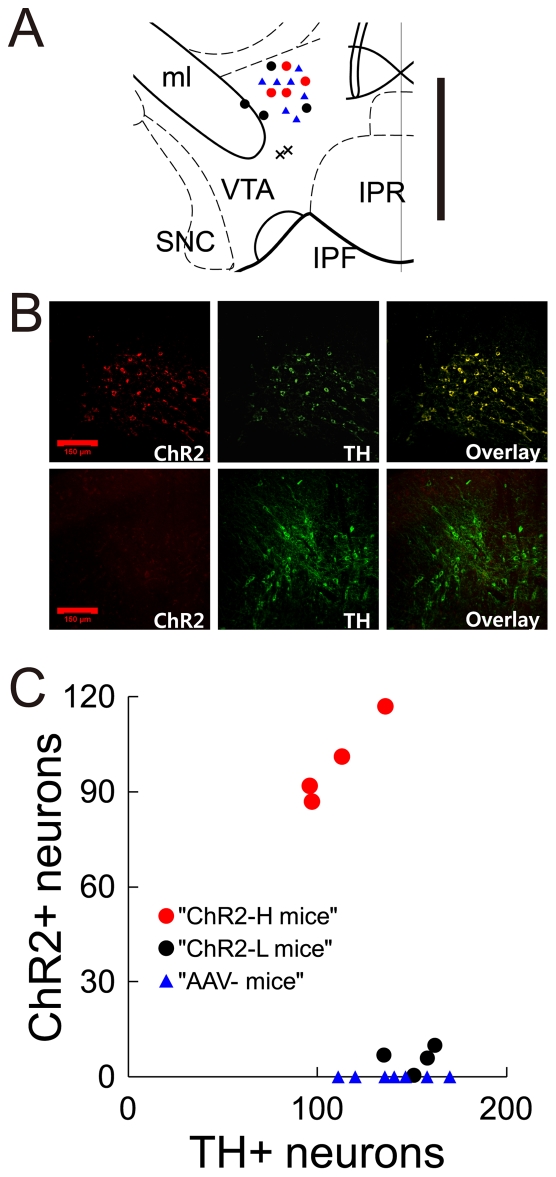
Expression of ChR2 in VTA dopamine neurons. **A**, Positions of viral injections in a coronal section of ventral midbrain (−3.28 mm from bregma). All injections were found to be within 0.06 mm rostral or caudal of this section. Each dot represents the injection site for an individual mouse (red circles for ChR2-H mice, black circles for ChR2-L mice, and blue triangles for AAV- mice). Vertical scale bar at right: 0.5 mm. **B**, Top, ChR2-tdTomato (red) colocalized with TH immunostaining (green) as shown in the overlay at right (yellow). Bottom, ChR2-tdTomato expression was not observed in another mouse. The center of these images corresponds to the ‘X’ marks in ‘A.’ Inset scale bars: 0.15 mm. **C**, Number of TH+ and ChR2+ neurons in each mouse. Based upon these results, mice were categorized as “ChR2-H” (red) or “ChR2-L” (black).

Channelrhodopsin-2 (ChR2) was introduced into the right VTA of dopamine transporter (DAT) IRES-Cre mutant mice [33] via injection of a Cre-dependent AAV-FLEX vector, which flanks the reversed ChR2 with sets of incompatible lox sites so that expression is localized to Cre-expressing cells (in this case, dopamine neurons) [34]. Of 8 mice in which histology was performed (4–5 weeks after injection of vector), all injection sites were found to be about 0.2 mm dorsal of the VTA (Fig. 1A). ChR2 (as indicated by tdTomato fluorescence) was colocalized with tyrosine hydroxylase (TH) immunostaining, with 98±6% (mean ± s.d.) of ChR2+ neurons being TH+ (Fig. 1B). All ChR2+ neurons were found to be in VTA, with none in SN. Because the expression levels of ChR2 appeared as two distinct clusters, we distinguished (post hoc) two subgroups of 4 mice each, which we designated as ChR2-H and ChR2-L, for mice expressing high and low levels of ChR2, respectively (Fig. 1C). In ChR2-H mice, 91±7% of TH+ neurons near the injection site also expressed ChR2, whereas only 17±4% of TH+ neurons expressed ChR2 in ChR2-L mice (possibly due to blockage of the injection cannula). ChR2-L mice effectively functioned as a blind control in behavioral experiments. Another control group (AAV- mice) consisted of 7 mice in which a cannula was implanted but no AAV vector was injected. In behavioral experiments, each of the three groups of mice received pulses of light directed towards VTA neurons, but only the ChR2-H group would be expected to display substantial optical activation of dopamine neurons.

Behavioral experiments commenced 14 days or more after injection of vector. A nose poke to the “active” port caused delivery (after a 100 ms delay) of a single, 200 ms square-wave pulse of blue light to the right VTA via an optic fiber (see Materials and Methods for the rationale in choosing this stimulation pattern). The precise temporal pattern of depolarization and action potentials that was induced in dopamine neurons is not known and is not critical to our hypothesis. The only critical point is that this excitation will increase firing rate within a temporal window of a few hundred milliseconds [16], [29], roughly similar to the naturally occurring activation of dopamine neurons (which is not highly stereotyped in its time course, but can vary over at least several hundred milliseconds in its duration depending on the nature of the reward-predictive stimulus [11], [35]; 200 ms is typical of the response to sudden onset of a familiar stimulus).

**Figure 2:**
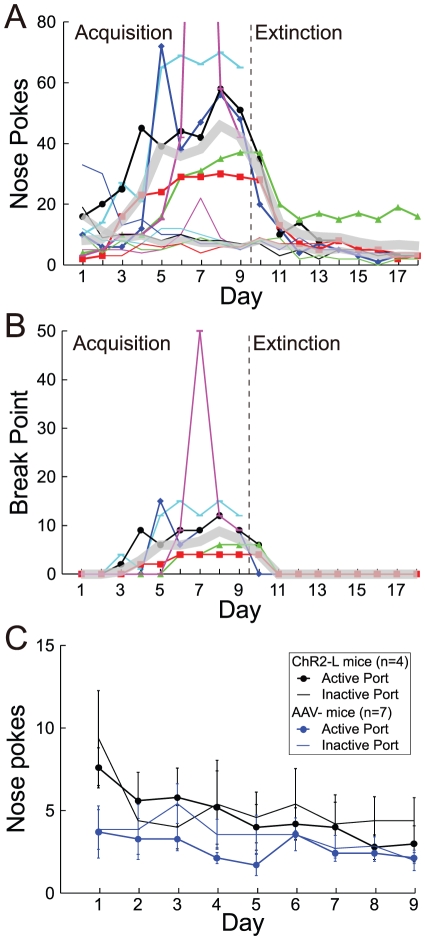
Operant responding for 200 ms of optical stimulation. **A**, Responses on the active (thick line) and inactive port (thin line) for each of the 4 ChR2-H mice, as well as 2 additional mice for which no histology results were obtained (magenta and cyan), over 9 days of acquisition followed by 9 days of extinction. On day 7, the mouse represented in magenta made 236 nose pokes at the active port (not shown). The thick gray lines, in ‘A’ and ‘B,’ represent the mean number of responses across the 4 ChR2-H mice. **B**, Break points on a PRS. Break points for the inactive port were zero in all cases and are not shown. **C**, Average responses (mean ± s.e.m.) on the active (thick line) and inactive (thin line) ports for ChR2-L (n = 4) and AAV- (n = 7) mice.

Each mouse was conditioned in an operant chamber over 9–18 days. After several days of conditioning, each of the four ChR2-H mice showed significant responding for optical stimulation (Fig. 2A) (Movie S1). On days 10–18, nose pokes were no longer followed by optical stimulation, and responding on the formerly active port declined in all mice (Fig. 2A). Two-way ANOVAs showed main effects of both day and port, as well as an interaction between the two, in each of the 4 mice (p<0.05; see “Statistical Analysis” in Materials and Methods). We also tested a total of 2 additional mice that had been injected with ChR2 but for which histological results could not be obtained. Both of these mice showed a significant increase in responding at the active port over 9 days of conditioning, equal to or greater than that in ChR2-H mice (Fig. 2A) (Two-way ANOVAs). However, they were not tested in extinction.

**Figure 3:**
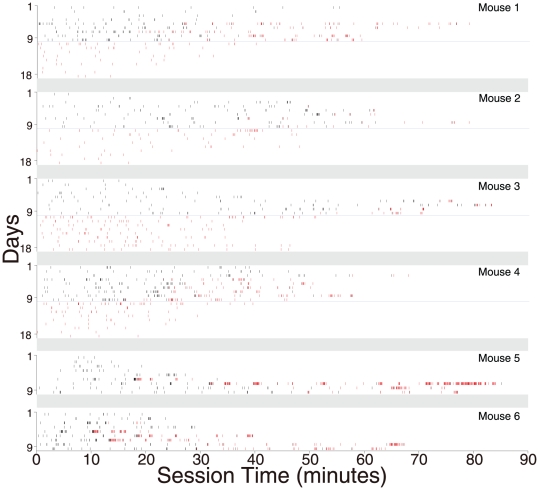
Rasters of response times during the operant task (90 minute sessions over 18 days in each of the 6 mice that displayed high levels of operant responding). This is the same data summarized in Fig. 2A,B. Responses that were followed by optical stimulation are in black, and those not followed by optical stimulation are in red. Blue horizontal lines divide the acquisition and extinction periods. The two mice shown at the bottom did not undergo the extinction phase, and no histology was performed in these mice.

In order to better quantify the reward value of the optical stimulation, we used a progressive ratio schedule (PRS) of reinforcement in which animals had to perform a larger number of responses for each additional pulse of light. The PRS was not administered in separate sessions, but rather in the same sessions described above. Each day (excluding extinction training on days 9–18), each of the first 20 nose pokes was followed by a pulse of light (a FR-1 schedule). Additional responses resulted in light pulses according to the PRS. Whereas figure 2A shows the total number of daily responses (on FR and PR schedules), figure 2B shows break points on the PRS (the largest number of responses made at the active port for a single pulse of light). Average break points (across days 6–9) across the 6 responding mice ranged from 4 to 20. The times of each operant response in each mouse, on both FR and PR schedules, are shown in figure 3.

In contrast to the ChR2-H mice, none of the ChR2-L or AAV- mice showed clear evidence of an increase in responding on the active port over 9 days of conditioning, nor did they show any difference in responding between active and inactive ports (Fig. 2C) (2-way ANOVAs in each mouse). To compare ChR2 mice (n = 8; both ChR2-H and ChR2-L) to AAV- controls (n = 7), we performed a two-way ANOVA in which group and nose-poke port (active versus inactive) were factors, with repeated measures across individual mice and across days 6–9. We found significant effects of both group and port, and an interaction between the two (p<0.05 in all 3 cases). Although we did not control for any potential effects of light that could be endowed by the AAV vector in the absence of ChR2, a review of the literature supported our view that such an effect would be extremely unlikely (Information S1).

**Figure 4:**
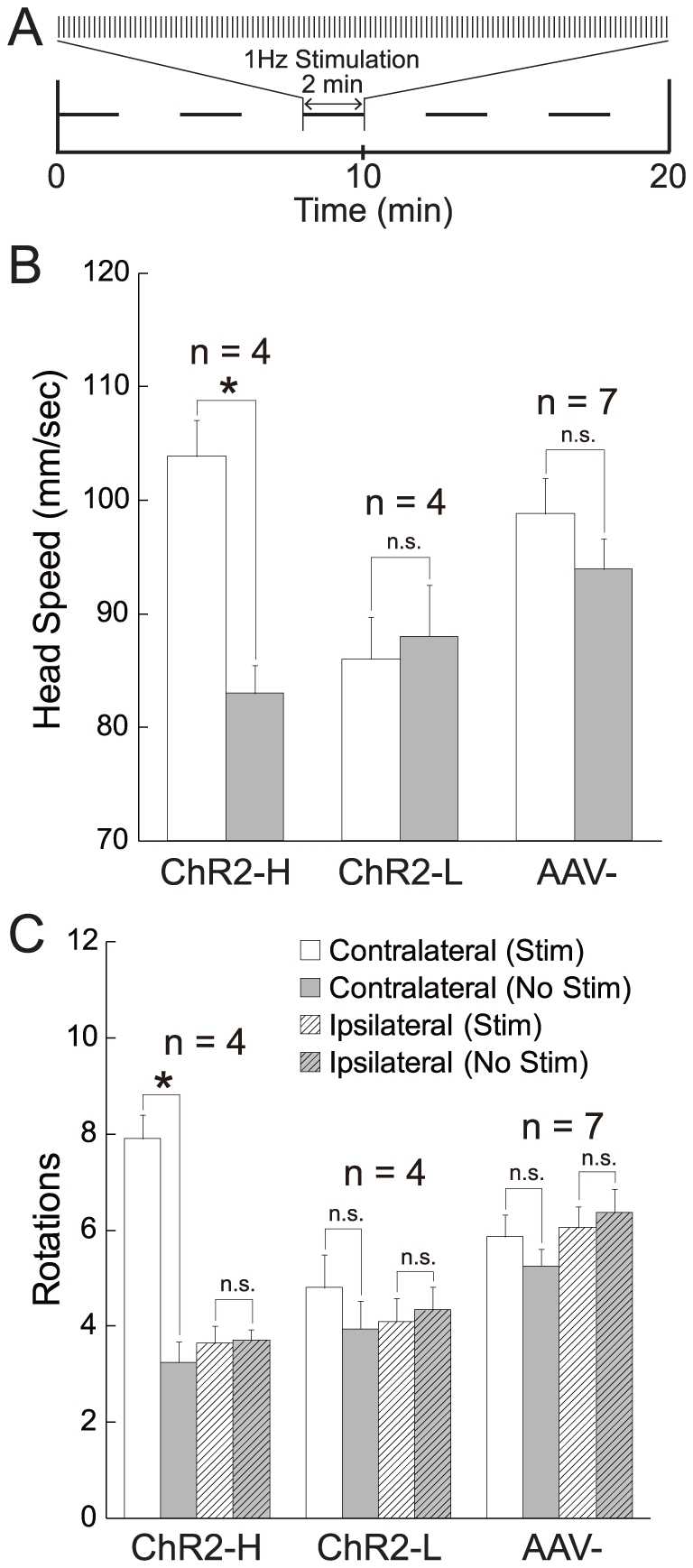
Optical stimulation promotes locomotion. **A**, Following 20 minutes of habituation, there were alternating periods of 2 minutes with and without stimulation (200 ms pulses at 1 Hz), for a total of 5 periods and 10 minutes of each condition. **B**, Both head speed and **C**, number of contralateral (but not ipsilateral) rotations were greater during stimulation (white) than non-stimulation (gray) in ChR2-H mice, but not in ChR2-L or AAV- mice.

In addition to its reinforcing effects, dopamine from VTA is also known to increase locomotion [15], and more specifically, contralateral rotation [36]. As an independent measure of the efficacy of optical stimulation in activating dopamine neurons, we measured locomotor behavior in separate sessions in which optical pulses of 200 ms were delivered once per second (Fig. 4A). Optical stimulation increased head speed (p<0.05 in each mouse, unpaired t-tests) (Fig. 4B) and contralateral rotations (p<0.01 across mice, paired t-test) (Fig. 4C) in ChR2-H mice but not in either of the control groups.

The responding observed in ChR2-H mice (Fig. 2A,B & Fig. 3) could conceivably be explained by the locomotor stimulant effects of dopamine, rather than through reinforcement. Mice perform nose pokes at a low rate even in the absence of reinforcement, and the optically induced dopamine release following a nose poke on the active port could drive additional nose pokes merely through a non-specific increase in locomotion. However, the enhanced responding on the active port was not paralleled by enhanced responding on the inactive port (Fig. 2A). Furthermore, conclusive evidence that optically induced locomotor stimulation cannot explain the responding comes from the fact that elevated responding on the active port was observed during the first few days of extinction training, in the complete absence of optical stimulation (Fig. 2A & Fig. 3).

**Figure 5:**
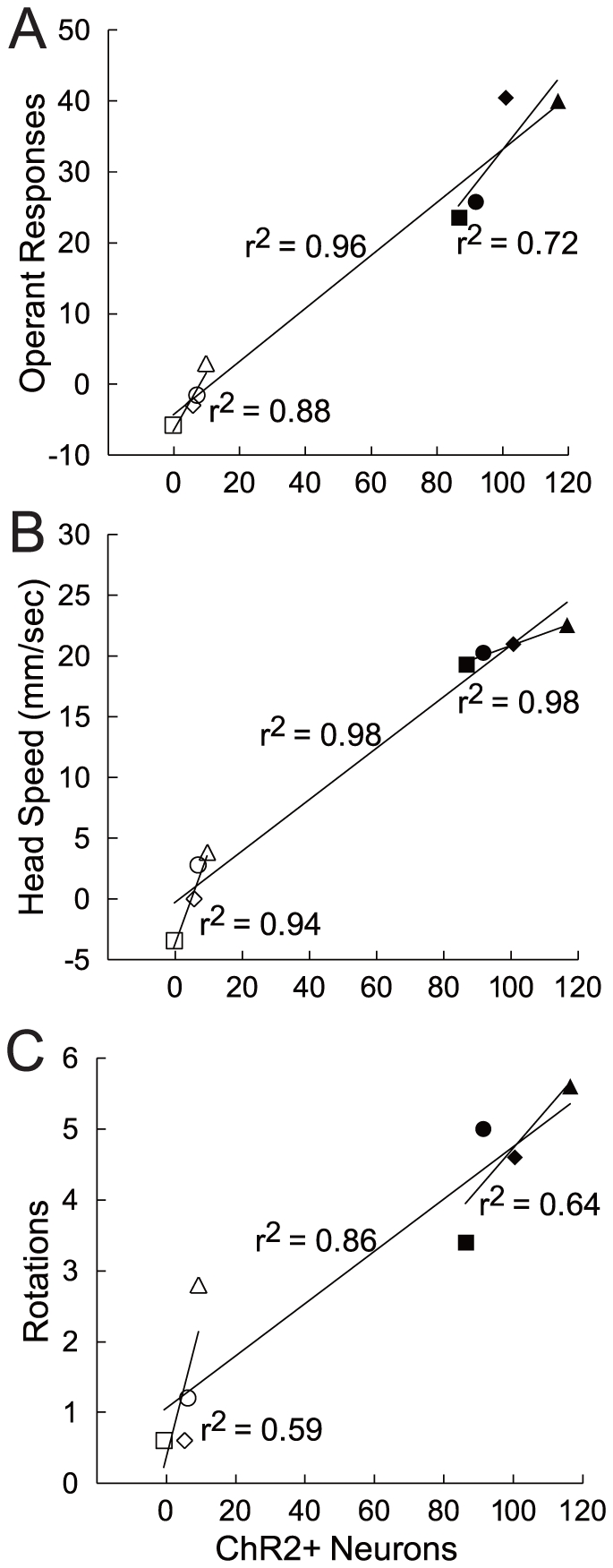
Behavioral responses correlate with the number of ChR2 positive neurons. Each point represents a single mouse. For each behavior, we measured the correlation for all ChR2 mice (long lines, p<0.01 for each behavior), as well as only the ChR2-H or ChR2-L mice (short lines). **A**, The y-axis indicates the difference in the number of operant responses at the active versus inactive ports (averaged across days 6–9; see Fig. 2A). **B**, Head speed. **C**, Rotations. In B and C, the y-axis indicates differences between stimulation and non-stimulation periods (see Fig. 4B,C).

The level of ChR2 expression across mice correlated strongly with each of the 3 behavioral responses (p<0.01 in each case) (Fig. 5). This is obvious when comparing responses between ChR2-H and ChR2-L mice in previous figures (Figs. 2 & 4). However, close inspection suggests a remarkably strong correlation even within each of these two subgroups of 4 mice, despite the low variance in ChR2 expression and behavioral responses within each subgroup (Fig. 5). We measured the correlation for each behavior in each subgroup (3 behaviors×2 subgroups = 6 potential correlations, with n = 4 in each case), and found that head speed showed significant correlations in both ChR2-H (r^2^ = 0.99, p = 0.01) and ChR2-L (r^2^ = 0.97, p = 0.03) mice (without correction for multiple comparisons) (Fig. 5B). Operant responding (Fig. 5A) and contralateral rotations (Fig. 5C) showed the same trend but did not reach significance (range between r^2^ = 0.77, p = 0.23 and r^2^ = 0.88, p = 0.06). There was also evidence of correlations across mice between the three behavioral responses in both ChR2-H and ChR2-L mice, as can be seen by comparing the responses of individual mice between the panels of figure 5. Indeed, a significant 3-way correlation was found between ChR2 expression, operant responding, and head speed in ChR2-H mice (r^2^ = 0.98, p = 0.01), and the same trend was found in ChR2-L mice (r^2^ = 0.85, p = 0.08).

These correlations suggests that despite the very small numbers of ChR2+ neurons in ChR2-L mice, optical stimulation may have elicited a small but behaviorally relevant activation of dopamine neurons. The more general inference that we make based on these correlations is that the number of ChR2 positive neurons is tightly linked to the magnitude of the optically-driven dopamine response, and that the magnitude of the dopamine response is tightly linked to behavior.

## Discussion

The three types of behaviors measured here (operant reinforcement, locomotion, and contralateral rotation) are all known to result from increases in dopamine transmission that are sustained over periods of minutes or longer (e.g. [1], [2], [15]). Given the strong correlations that we observed between ChR2 expression and all three behavioral responses (Fig. 5), the present results provide compelling evidence that the brief, naturally occurring activation of dopamine neurons is sufficient for positive reinforcement of preceding actions, as hypothesized by RL models of dopamine function [13], [14]. In addition, these results appear inconsistent with the hypotheses that the brief activation of dopamine neurons does not convey a functional reward signal [18], [19], and that it does not directly drive learning [23], [24].

The optically driven reinforcement of operant responding observed here is comparable to the electrically driven “brain stimulation reward” (BSR) described first by Olds and Milner [37]. Electrical BSR elicited from certain brain regions (and with certain stimulation protocols that have been refined over the years to produce maximal reward effects) can have extremely high reward value, far greater than that of natural rewards [38], [39]. The response rates of our mice for optical BSR were low relative to the very high response rates that are commonly observed for electrical BSR of the lateral hypothalamus. However, when optical BSR was delivered on a progressive ratio schedule, we observed average break points (across days) ranging from 4 to 20 across our 6 mice. These break points are similar to those observed with electrical BSR of lateral hypothalamus, which have been found to range between 5 and 25 across studies [40]–[45]. The strength of our optical BSR was almost certainly limited by the fact that our stimulation was unilateral, and would only be expected to cause dopamine release in the ipsilateral hemisphere [46]. By contrast, the standard means of evoking electrical BSR, and the one used in these papers, is unilateral stimulation of lateral hypothalamus, which has been found to cause bilateral dopamine release in nucleus accumbens, both with microdialysis [47] and voltametry (Cossette and Shizgal, 2011 SFN abstract). The naturally occurring activation of dopamine neurons occurs bilaterally, in both VTA and SN. Thus the optical BSR described here appeared to have moderate reward value, and its value could probably be increased further through stimulation that is bilateral and includes SN as well as VTA.

Adamantidis and colleagues have recently tested the effect of optical activation of dopamine neurons on operant responding in mice, using an optogenetic technique virtually identical to that used here [28]. They used stimulation parameters that they found to be near optimal in evoking maximal dopamine release in the nucleus accumbens (20 pulses of 5 ms at 25 Hz, 0.8 s total stimulation period). They found that mice preferred food plus optical stimulation over food alone. In a similar study, it was observed that mice preferred water plus 1.0 s of optical stimulation to water alone [30]. However, despite the fact that the total stimulus duration used by Adamantidis and colleagues was four times longer than ours (0.8 s versus 0.2 s), and that of a natural reward response in dopamine neurons, they found that it was not sufficient for reinforcement in and of itself [28]. There are numerous potential explanations for their negative result, one being that they only examined the effect optical stimulation alone following extinction of responding for food, so that a modest rewarding effect of stimulation may have been overshadowed by the “disappointment” (and negative RPE) of not receiving food following operant responses. Regardless of the explanation, our positive result provides strong evidence that activation of dopamine neurons alone is sufficient for positive reinforcement.

Our results are complemented by those of Zweifel and colleagues [48], who selectively disrupted NMDA-type glutamate receptors in dopamine neurons and found that mice were impaired in their acquisition of a variety of reinforced behaviors. Since it is likely that the natural excitation of dopamine neurons following reward events is substantially mediated by NMDA receptors, their results suggest that the transient activation of dopamine neurons contributes to the positive reinforcement of natural reward stimuli.

Although the present results demonstrate that the transient activation of dopamine neurons is sufficient for positive reinforcement, it does not appear to be necessary for reinforcement [24]–[26]. Reinforcement is likely too important to depend entirely on any one type of neuron, and indeed, many types of neurons throughout a large portion of the brain have been found to carry information about reward, and to signal various sorts of RPE. It will therefore be important to characterize exactly what sort of reinforcement is mediated by dopamine. Since dopamine neurons innervate many brain regions, the particular “object” of reinforcement (stimuli versus actions, or general plans for behavior versus specific motor plans) can be expected to differ across brain regions. In the well studied case of the nucleus accumbens, dopamine appears to be important, and possibly necessary, for learning the “incentive motivational value” of stimuli and actions so as to guide an animal's behavior in a fast and “automated” fashion [1].

It has long been thought that that some drugs are addictive because they mimic the natural reward signal of dopamine neurons [2], [49]. However, whereas the natural dopamine signal (dopamine elevation in target areas) is on the scale of a few seconds, the duration of drug effects is on the scale of tens of minutes (thousands of seconds). By closing the temporal gap between studies of drug and natural reward, the present results provide a key piece of evidence in support of the view that addictive drugs drive reinforcement by mimicking the natural reward signal of dopamine neurons. Dopamine appears to be sufficient for reinforcement whether it is increased by a natural reward for only a second, or by a drug for many minutes.

## Methods

### Animals and Ethics Statement

Male dopamine transporter (DAT) IRES-Cre mice [33] were obtained from Jackson Laboratory (Bar Harbor, ME, USA). All procedures were in accordance with the NIH Guide for Care and Use of laboratory animals, and were approved by the Animal Care and Use Committee of Massachusetts Institute of Technology (protocol number 0110-007-13).

### Virus Preparation and Injection

We used an adeno-associated virus (AAV-FLEX-ChR2-tdTomato) to drive Cre-dependent expression of Channelrhodopsin-2 (ChR2) [34]. Replication-incompetent AAV-Flex-ChR2-tdTomato, serotype 8, was manufactured from the AAV-Flex-ChR2-tdTomato plasmid (Addgene plasmid 18917, available at http://www.addgene.org/pgvec1?f=c&cmd=findpl&identifier=18917 fromthe University of North Carolina Gene Therapy Vector Core). AAV-Flex-ChR2-tdTomato virus (1 µl, 4.5×10^12^/µL) was injected through a cannula into the right VTA (3.28 mm caudal and 4.4 mm ventral of bregma; 0.48 mm lateral) over a 10 minute period. Behavioral experiments began 14 or more days following injection of virus.

### Optical Stimulation

A 100 mW, 473 nm blue laser (BL473TC-100, Shanghai Laser & Optics Century Co., Ltd.) was coupled to a 200 µm-diameter optical fiber (BFH48-200, Thorlabs) through a rotary joint (Doric Lenses). Light irradiance, as measured with an 818-SL photodetector (Newport Co.), was 477 mW/mm2 at the fiber tip, within the typical range of light intensities used for brief ChR2 activation. The optic fiber was inserted into the brain through the same cannula that was used for injection of virus. We found no evidence of any behavioral effects of light in the absence of ChR2 (Fig. 2C, Fig. 4B, 4C).

### Choice of Stimulation Pattern

The duration of 200 ms for the pulse of light was chosen to mimic the duration of the increase in firing rate of dopamine neurons that is observed following the sudden onset of a natural reward event (e.g. [4]). The particular shape of the stimulation (a single square wave) is presumably not of great significance for the issues of interest here, but it was chosen to roughly mimic the depolarizing current across the membrane of dopamine neurons that may result from excitation of glutamate receptors, and particularly NMDA receptors, shortly after the onset of a reward event. It is reasonable to suspect that a reward event is followed by a brief, high frequency train of action potentials in glutamatergic afferents to midbrain dopamine neurons. Such a pattern of activity in glutamatergic afferents is known to cause a relatively sustained, NMDA receptor-mediated EPSP in dopamine neurons in vitro, which elicits a burst of several action potentials [50]. Thus a 200 ms depolarization mediated by sustained optical activation of ChR2 may cause a burst of several action potentials in dopamine neurons, similar to that observed in vivo in response to natural rewards. However, the number and pattern of evoked action potentials is unknown. The only assumption that is critical to our interpretation of the results is that some additional action potentials, beyond the background rate, were elicited by optical stimulation, and that these occurred within a few hundred milliseconds after each operant response.

The short delay of 100 ms between detection of a nose poke and onset of optical reward was chosen to maximize the speed and efficacy of reinforcement. Longer delays would be expected to result in slower acquisition of operant responding, and after acquisition, a lower asymptotic reward value due to temporal discounting. It is noteworthy that 100 ms is also the approximate delay between onset of a reward-predicting sensory stimulus and onset of the increase in firing rate of dopamine neurons (e.g. [4]).

### Behavior

Operant responding was measured in separate sessions in a chamber that was dark except for light emanating from each of two nose poke ports (Movie S1). Inside each port was a small red LED that extinguished at the onset of a nose poke in either the active or inactive ports, and remained off for 300 ms. Within each daily session, the first 20 nose pokes were followed by optical stimulation (a FR-1 schedule). Optical stimulation commenced 100 ms after detection of a nose poke at the active port and lasted for 200 ms (a single square wave). Thus, onset of the red light coincided with the offset of optical stimulation, 300 ms after detection of a nose poke. The port then became immediately available for another nose poke. Thus a mouse could receive a maximum of 1 optical pulse every 300 ms (when working on a FR1 schedule), although no mice were observed to approach this limit. Mice were placed in the operant chamber for at least 1 hour each day. After 1 hour, a session ended when the mouse failed to make any response for 5 minutes. The longest session was 1.5 hours.

If a mouse completed 20 nose pokes within one session, for the remainder of the session a progressive ratio schedule (PRS) was applied in which a larger number of nose pokes were required for each additional pulse of optical stimulation. The schedule was 1, 2, 4, 6, 9, 12, 15, 20, 25, 32, 40 and 50 nose pokes, with 50 being the maximum break point that we observed across all sessions in all mice. Our use of the PRS may have quantitively altered responding on the FR-1 schedule (on the subsequent day following the PRS), but any such alteration is not a primary concern given that our interest is simply in determining whether or not the dopamine RPE is sufficient to drive reinforcement.

Locomotor responses to optical stimulation were measured in separate behavioral sessions. Both head speed and rotations were tracked with a Noldus EthoVision automated tracking system. Mice were habituated to the behavior chamber for 20 minutes. In the following 20 minutes, there were alternating periods of 2 minutes of optical stimulation followed by 2 minutes without stimulation, for a total of 5 periods and 10 minutes of each condition (Fig. 4A). During each 2 minute stimulation period, optical square-wave pulses of 200 ms duration were delivered once per second. Whereas figures 4B and 4C show locomotor activity in both stimulation and non-stimulation periods, figures 5B and 5C show differences between the full 10 minutes of stimulation versus the 10 minutes without stimulation.

### Histology and Immunofluorescence

The counts of TH-positive and ChR2-positive neurons (Fig. 1C) were performed in 7 neighboring coronal slices of 30 µm each, taken from just below the center of the lesion caused by the guide cannula (200 µm diameter). ChR2-positive neurons were identified by co-expression of the fluorescent protein tdTomato, whereas TH-positive neurons were identified based on standard immunostaining techniques. Only regions of red or green fluorescence larger than 17 µm in diameter were counted as neurons. ChR2-L and AAV- mice had low levels of red fluorescence. For this reason, the gain and exposure time was increased in order to identify any potential tdTomato that might be present. Under these conditions, small regions (∼5 µm) of weak auto-fluorescence were visible (Fig. 1B, lower left panel). That this was auto-fluorescence, and not tdTomato, was evident in the fact that similar levels and patterns of red fluorescence were visible in AAV- mice, which had not received any ChR2-tdTomato-containing virus.

We did not find evidence of any ChR2-tdTomato expression in SN, but we do not exclude the possibility that some SN dopamine neurons may have expressed ChR2 and may have contributed to behavioral responses. Although we targeted dopamine neurons of the VTA, it was not the purpose of this study to distinguish VTA from SN.

### Statistical Analyses

Statistical significance was defined as p<0.05 in all tests. Operant responses in individual mice (Fig. 2A) were analyzed with a two-way ANOVA, with day and nose poke port (active versus inactive) as the two factors. Tukey's test was used to test for an interaction. For comparison between ChR2 and AAV- groups of mice, we performed a two-way ANOVA in which group and nose-poke port (active versus inactive) were factors, with repeated measures across individual mice and across days 6–9. For comparing the locomotor responses of figure 4B and 4C across populations of mice, paired t-tests were performed between locomotor activity during stimulation versus non-stimulation periods. Within each individual mouse, the significance of the effect of optical stimulation was determined by unpaired t-tests of locomotion between the five stimulation versus non-stimulation periods. Pearson's correlation coefficient (r^2^) was calculated to measure correlations.

## Supporting Information

Movie S1 **A mouse working for optical stimulation on a FR1 schedule.** Light can be seen emanating from both the active and inactive ports.
(MOV)

Information S1 **We reviewed the literature to investigate the possibility that our AAV vector might confer light sensitivity in the absence of ChR2 expression.** We cited 29 studies that have used AAV vector in combination with optical stimulation of neural tissue, and we categorized these using a Venn diagram. The text reads “In the present work, we controlled for nonspecific effects of light, both in our AAV- group and by demonstrating a strong correlation of behavioral responses with ChR2 expression. We did not control for the possibility that AAV might confer light sensitivity in the absence of ChR2 expression. While this is certainly possible, we consider it extremely unlikely given basic knowledge of photochemistry and membrane excitability. In addition, the technique of optical stimulation in combination with AAV vector is no longer so new. To investigate the available data on this issue, we have reviewed a total of 29 papers that have used AAV for optogenetic experiments, including 6 papers on dopamine neurons. 13 of these 29 papers included AAV control experiments (3 of 6 papers on dopamine neurons). None of the 13 papers found any effect of light in AAV controls. 16 of the 29 papers did not include any AAV controls. We note that most of the papers without AAV controls were published in well respected journals, suggesting that many reviewers were not particularly concerned about the lack of AAV controls.”
(PDF)
