# Supplementary material for: Optogenetic Mimicry of the Transient Activation of Dopamine Neurons by Natural Reward Is Sufficient for Operant Reinforcement
Source: PLoS One. 2012 Apr 10;7(4):e33612. doi: 10.1371/journal.pone.0033612 (PMC3323614; doi:10.1371/journal.pone.0033612)
Supplement: Information S1 — We reviewed the literature to investigate the possibility that our AAV vector might confer light sensitivity in the absence of ChR2 expression. We cited 29 studies that have used AAV vector in combination with optical stimulation of neural tissue, and we categorized these using a Venn diagram. The text reads “In the present work, we controlled for nonspecific effects of light, both in our AAV- group and by demonstrating a strong correlation of behavioral responses with ChR2 expression. We did not control for the possibility that AAV might confer light sensitivity in the absence of ChR2 expression. While this is certainly possible, we consider it extremely unlikely given basic knowledge of photochemistry and membrane excitability. In addition, the technique of optical stimulation in combination with AAV vector is no longer so new. To investigate the available data on this issue, we have reviewed a total of 29 papers that have used AAV for optogenetic experiments, including 6 papers on dopamine neurons. 13 of these 29 papers included AAV control experiments (3 of 6 papers on dopamine neurons). None of the 13 papers found any effect of light in AAV controls. 16 of the 29 papers did not include any AAV controls. We note that most of the papers without AAV controls were published in well respected journals, suggesting that many reviewers were not particularly concerned about the lack of AAV controls.” (PDF) [file pone.0033612.s002.pdf]

Supporting Information S1

Review of Literature with Respect to AAV Controls in Optogenetic Experiments

In the present work, we controlled for nonspecific effects of light, both in our AAV- group and by demonstrating a strong correlation of behavioral responses with ChR2 expression. We did not control for the possibility that AAV might confer light sensitivity in the absence of ChR2 expression. While this is certainly possible, we consider it extremely unlikely given basic knowledge of photochemistry and membrane excitability. In addition, the technique of optical stimulation in combination with AAV vector is no longer so new. To investigate the available data on this issue, we have reviewed a total of 29 papers that have used AAV for optogenetic experiments, including 6 papers on dopamine neurons. 13 of these 29 papers included AAV control experiments (3 of 6 papers on dopamine neurons). None of the 13 papers found any effect of light in AAV controls. 16 of the 29 papers did not include any AAV controls. We note that most of the papers without AAV controls were published in well respected journals, suggesting that many reviewers were not particularly concerned about the lack of AAV controls.

Below we provide these 29 references. In addition and we have categorized the papers within a Venn diagram, in which we have distinguished papers with AAV control versus no AAV control, papers on dopamine neurons versus other neurons, and in vivo versus in vitro.

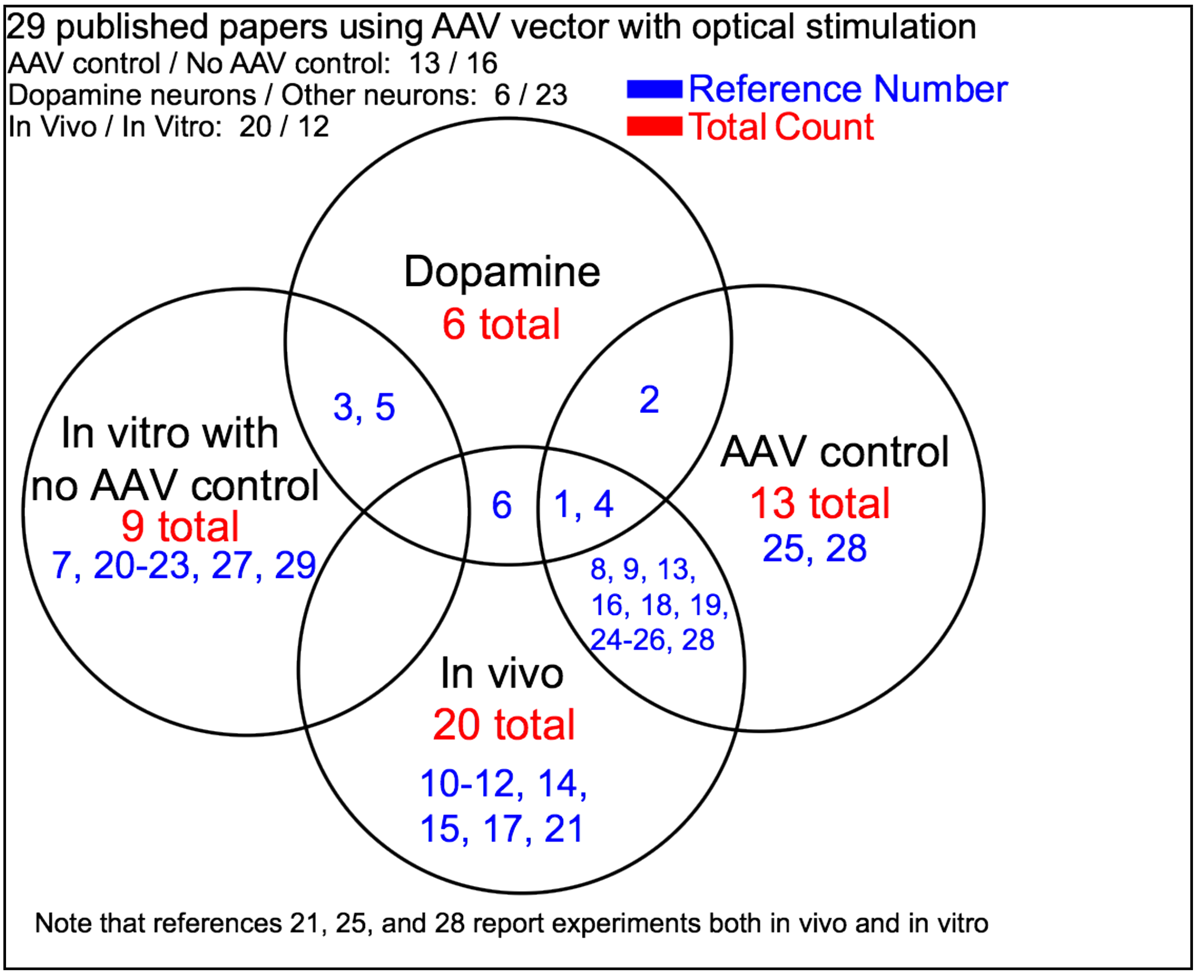

Studies that used AAV as a vector in combination with optical stimulation

1. Adamantidis AR, Tsai HC, Boutrel B, Zhang F, Stuber GD, et al. (2011) Optogenetic interrogation of dopaminergic modulation of the multiple phases of reward-seeking behavior. J Neurosci 31: 10829-10835.
2. Brown MT, Bellone C, Mameli M, Labouebe G, Bocklisch C, et al. (2010) Drug-driven AMPA receptor redistribution mimicked by selective dopamine neuron stimulation. PLoS One 5: e15870.
3. Stuber GD, Hnasko TS, Britt JP, Edwards RH, Bonci A (2010) Dopaminergic terminals in the nucleus accumbens but not the dorsal striatum corelease glutamate. J Neurosci 30: 8229-8233.
4. Tsai HC, Zhang F, Adamantidis A, Stuber GD, Bonci A, et al. (2009) Phasic firing in dopaminergic neurons is sufficient for behavioral conditioning. Science 324: 1080-1084.
5. Tecuapetla F, Patel JC, Xenias H, English D, Tadros I, et al. (2010) Glutamatergic signaling by mesolimbic dopamine neurons in the nucleus accumbens. J Neurosci 30: 7105-7110.
6. Domingos AI, Vaynshteyn J, Voss HU, Ren X, Gradinaru V, et al. (2011) Leptin regulates the reward value of nutrient. Nat Neurosci 14: 1562-1568.
7. Nagode DA, Tang AH, Karson MA, Klugmann M, Alger BE (2011) Optogenetic Release of ACh Induces Rhythmic Bursts of Perisomatic IPSCs in Hippocampus. PLoS One 6: e27691.
8. Carter ME, Yizhar O, Chikahisa S, Nguyen H, Adamantidis A, et al. (2010) Tuning arousal with optogenetic modulation of locus coeruleus neurons. Nat Neurosci 13: 1526-1533.
9. Johansen JP, Hamanaka H, Monfils MH, Behnia R, Deisseroth K, et al. (2010) Optical activation of lateral amygdala pyramidal cells instructs associative fear learning. Proc Natl Acad Sci U S A 107: 12692-12697.
10. Lee JH, Durand R, Gradinaru V, Zhang F, Goshen I, et al. (2010) Global and local fMRI signals driven by neurons defined optogenetically by type and wiring. Nature 465: 788-792.
11. Cardin JA, Carlen M, Meletis K, Knoblich U, Zhang F, et al. (2009) Driving fast-spiking cells induces gamma rhythm and controls sensory responses. Nature 459: 663-667.
12. Sohal VS, Zhang F, Yizhar O, Deisseroth K (2009) Parvalbumin neurons and gamma rhythms enhance cortical circuit performance. Nature 459: 698-702.
13. Bi A, Cui J, Ma YP, Olshevskaya E, Pu M, et al. (2006) Ectopic expression of a microbial-type rhodopsin restores visual responses in mice with photoreceptor degeneration. Neuron 50: 23-33.
14. Zhang Y, Ivanova E, Bi A, Pan ZH (2009) Ectopic expression of multiple microbial rhodopsins restores ON and OFF light responses in retinas with photoreceptor degeneration. J Neurosci 29: 9186-9196.
15. Haubensak W, Kunwar PS, Cai H, Ciocchi S, Wall NR, et al. (2010) Genetic dissection of an amygdala microcircuit that gates conditioned fear. Nature 468: 270-276.
16. Busskamp V, Duebel J, Balya D, Fradot M, Viney TJ, et al. (2010) Genetic reactivation of cone photoreceptors restores visual responses in retinitis pigmentosa. Science 329:

413-417.
17. Ciocchi S, Herry C, Grenier F, Wolff SB, Letzkus JJ, et al. (2010) Encoding of conditioned fear in central amygdala inhibitory circuits. Nature 468: 277-282.
18. Witten IB, Lin SC, Brodsky M, Prakash R, Diester I, et al. (2010) Cholinergic interneurons control local circuit activity and cocaine conditioning. Science 330: 1677-1681.
19. Tye KM, Prakash R, Kim SY, Fenno LE, Grosenick L, et al. (2011) Amygdala circuitry mediating reversible and bidirectional control of anxiety. Nature 471: 358-362.
20. Gu Z, Yakel JL (2011) Timing-dependent septal cholinergic induction of dynamic hippocampal synaptic plasticity. Neuron 71: 155-165.
21. Carlen M, Meletis K, Siegle JH, Cardin JA, Futai K, et al. (2011) A critical role for NMDA receptors in parvalbumin interneurons for gamma rhythm induction and behavior. Mol Psychiatry (advanced online publication; doi:10.1038/mp.2011.31).
22. Gunaydin LA, Yizhar O, Berndt A, Sohal VS, Deisseroth K, et al. (2009) Ultrafast optogenetic control. Nat Neurosci 13: 387-392.
23. Kleinlogel S, Terpitz U, Legrum B, Gokbuget D, Boyden ES, et al. (2011) A gene-fusion strategy for stoichiometric and co-localized expression of light-gated membrane proteins. Nat Methods 8: 1083-1088.
24. Stuber GD, Sparta DR, Stamatakis AM, van Leeuwen WA, Hardjoprajitno JE, et al. (2011) Excitatory transmission from the amygdala to nucleus accumbens facilitates reward seeking. Nature 475: 377-380.
25. Lobo MK, Covington HE, 3rd, Chaudhury D, Friedman AK, Sun H, et al. (2010) Cell type-specific loss of BDNF signaling mimics optogenetic control of cocaine reward. Science 330: 385-390.
26. Kravitz AV, Freeze BS, Parker PR, Kay K, Thwin MT, et al. (2010) Regulation of parkinsonian motor behaviours by optogenetic control of basal ganglia circuitry. Nature 466: 622-626.
27. Ellender TJ, Huerta-Ocampo I, Deisseroth K, Capogna M, Bolam JP (2011) Differential modulation of excitatory and inhibitory striatal synaptic transmission by histamine. J Neurosci 31: 15340-15351.
28. Pascoli V, Turiault M, Luscher C (2011) Reversal of cocaine-evoked synaptic potentiation resets drug-induced adaptive behaviour. Nature 481: 71-75.
29. Atasoy D, Aponte Y, Su HH, Sternson SM (2008) A FLEX switch targets Channelrhodopsin-2 to multiple cell types for imaging and long-range circuit mapping. J Neurosci 28: 7025-7030.
